# Analyses of *ATP7B* mRNA in Nasopharyngeal Swab Samples Increase Yields of Wilson Disease Molecular Genetic Diagnostics

**DOI:** 10.1155/humu/8416660

**Published:** 2026-02-25

**Authors:** Lenka Steiner Mrázová, Alena Vrbacká, Filip Majer, Viktor Stránecký, Lenka Nosková, Daniela Záhoráková, Jitka Májovská, Ibrahim Bitar, Jiří Klempíř, Jana Šaligová, Stella Majlingová, Mária Giertlová, Petra Drenčáková, Denisa Harvanová, Pavla Solařová, Radan Brůha, Petr Dušek, Stanislav Kmoch, Jakub Sikora, Ivana Jedličková

**Affiliations:** ^1^ Research Unit for Rare Diseases, Department of Paediatrics and Inherited Metabolic Disorders, First Faculty of Medicine, Charles University and General University Hospital in Prague, Prague, Czech Republic, cuni.cz; ^2^ Department of Paediatrics and Inherited Metabolic Disorders, First Faculty of Medicine, Charles University and General University Hospital in Prague, Prague, Czech Republic, cuni.cz; ^3^ Department of Microbiology, Faculty of Medicine and University Hospital Pilsen, Charles University, Pilsen, Czech Republic, cuni.cz; ^4^ Biomedical Center, Faculty of Medicine, Charles University, Pilsen, Czech Republic, cuni.cz; ^5^ Department of Neurology and Center of Clinical Neuroscience, First Faculty of Medicine, Charles University and General University Hospital in Prague, Prague, Czech Republic, cuni.cz; ^6^ Department of Paediatrics and Adolescent Medicine, Faculty of Medicine, P. J. Šafárik University and Children′s Faculty Hospital Košice, Košice, Slovakia; ^7^ Department of Neurology, Faculty of Medicine, P. J. Šafárik University, Košice, Slovakia, upjs.sk; ^8^ Department of Clinical Neurosciences, Center of Clinical and Preclinical Research MEDIPARK, P. J. Šafárik University, Košice, Slovakia, upjs.sk; ^9^ Outpatient Clinic of Medical Genetics, Unilabs, Košice, Slovakia, unilabs.pt; ^10^ Associated Tissue Bank, Faculty of Medicine, P.J. Šafárik University and L. Pasteur University Hospital in Košice, Košice, Slovakia; ^11^ Department of Medical Genetics, University Hospital Hradec Králové, Hradec Králové-Nový, Czech Republic, fnhk.cz; ^12^ 4th Department of Internal Medicine, First Faculty of Medicine, Charles University and General University Hospital in Prague, Prague, Czech Republic, cuni.cz; ^13^ Department of Radiology, First Faculty of Medicine, Charles University and General University Hospital in Prague, Prague, Czech Republic, cuni.cz; ^14^ Institute of Pathology, First Faculty of Medicine, Charles University and General University Hospital in Prague, Prague, Czech Republic, cuni.cz

**Keywords:** *ATP7B*, long-read amplicon sequencing, mRNA splicing, nasopharyngeal swab, synonymous variants, Wilson disease

## Abstract

Wilson disease (WD) is an autosomal recessive disorder of copper transport caused by bi‐allelic pathogenic variants in the ATPase copper transporting beta gene (*ATP7B*). Results of standard genetic diagnostics remain inconclusive in 3%–20% of WD patients in part due to problematic assessment of variants of unknown or conflicting pathogenicity (synonymous variants included). Correct interpretation of potential effects of such variants can be substantially enhanced by RNA analyses. This strategy is, however, of limited utility in WD patients because of predominant liver expression of *ATP7B*. To avoid invasive bioptic liver collection and increase WD diagnostic yields, we searched for a surrogate tissue sample and identified profiles of *ATP7B* transcripts in nasopharyngeal swabs that were comparable to liver. Amplicons spanning *ATP7B* Exons 3–21 were prepared from the swab material and analysed by long‐read nanopore sequencing to enable the detection of splicing changes and variant phasing. Diagnostic utility of this novel *in vivo* methodology was demonstrated by characterization of mRNA splicing abnormalities caused by synonymous *ATP7B* variants c.1488C>T (p.(Gly496=)), c.2241C>T (p.(Ile747=)), c.2292C>T (p.(Phe764=)), and a nonsense variant c.2336G>A (p.(Trp779Ter)) in four WD patients, who were not genetically resolved by standard techniques. Nasopharyngeal swab sampling is minimally invasive and allows effective analyses of mRNA to detect and/or validate effects of *ATP7B* variants in WD patients. Conclusive genetic diagnosis attained by this novel technique may facilitate family counselling and substantiate initiation of copper‐chelation therapy in presymptomatic individuals.

## 1. Introduction

Wilson disease (WD, OMIM 606882) is a rare autosomal recessive disorder of intracellular copper trafficking resulting in its abnormal accumulation, particularly in the liver and brain [1, 2].

WD diagnostics are challenging due to variable age at onset and diverse manifestations in different organ systems [3]. Patients commonly present with liver disease (hepatitis, cirrhosis, and acute liver failure), neurological and/or psychiatric symptoms (Parkinsonism, dystonia, dysarthria, depression, and personality changes). Kidney disease [4], osteoarticular [5], endocrine [6], or myocardial [7, 8] pathologies may also develop.

WD diagnostic algorithm builds on Leipzig score that combines clinical (neurological) evaluation, Kayser–Fleischer corneal ring assessment, biochemical findings (low serum ceruloplasmin concentrations, high serum nonceruloplasmin‐bound copper, increased urinary copper excretion and increased liver copper, hematological testing), brain magnetic resonance imaging, and molecular genetic analyses [1, 9].

Genetically, WD is caused by biallelic variants in the ATPase copper transporting beta gene (*ATP7B*, OMIM 606882). Currently, there are 1000 pathogenic or likely pathogenic variants reported in *ATP7B* in the VarSome data aggregator [10] (VarSome & VarSome Clinical v.13.11.0), including single‐nucleotide variants (SNVs), small deletions or insertions [11], large intragenic deletions mediated by *Alu* repeat elements [12], and noncoding variants affecting mRNA synthesis/processing [13–15]. Most of the variants in the coding region of *ATP7B* are easily identifiable by routine genetic testing of genomic DNA (gDNA) isolated from peripheral white blood cells (WBCs).

WD therapy builds on long‐term copper chelation protocols in symptomatic patients. Importantly, these treatments are also suggested to presymptomatic individuals carrying biallelic pathogenic *ATP7B* variants [9].

Genetic diagnosis remains inconclusive in 3%–20% of WD patients with only one or no *ATP7B* pathogenic variant detected [12, 16–21]. Synonymous variants affecting *ATP7B* mRNA splicing have also been previously reported [22–26]. An *in vivo* evaluation of the effects of these variants on mRNA synthesis and processing has, so far, used invasive bioptic liver sampling. Alternative approaches include *in silico* splicing predictions, mRNA analyses in skin fibroblasts [22] or WBCs with minimal *ATP7B* expression or minigene splicing assays [23–26]. All these approaches are either laborious, need invasive sample collection, or do not allow to effectively determine the phase of the variants.

To overcome these limitations, we searched for an alternative and easily attainable clinical sample with sufficient *ATP7B* mRNA expression that would allow *in vivo* testing of the effects of putative splicing variants in WD patients. Herein, we document that *ATP7B* mRNA is abundantly expressed in nasopharyngeal swabs and demonstrate that long‐read sequencing of an amplicon of *ATP7B* cDNA, prepared from this material, substantially facilitates WD molecular genetic diagnostics.

## 2. Materials and Methods

Four WD patients (P1–P4), with incomplete genetic diagnosis, were included in this study. The diagnosis of WD was established based on characteristic clinical and biochemical findings together with a Leipzig score over 4. No liver disease was reported among the family members of any of the patients. Clinical characteristics of the tested individuals are summarized in Table 1.

**Table 1:** Clinical characteristics of the patients. The initial suspicion of WD was based on the neurological presentation in P1 and accidental biochemical identification of liver dysfunction in P2, P3, and P4. Cut‐off for abnormal findings: ceruloplasmin < 0.2 g/L; 24 h urine Cu excretion > 1.6 * μ*mol/L; liver copper > 250 * μ*g/g. AAO, age at onset; ALT, alanine transaminase; AST, aspartate transaminase; CPL, ceruloplasmin; Cu, copper; IBS, irritable bowel syndrome; K‐F ring, Keyser–Fleischer ring; MRI, magnetic resonance imaging; ULN, upper limit of norm.

**Patient ID**	**AAO (yrs)**	**Initial suspicion to WD**	**K-F ring**	**Presenting symptoms**	**MRI (brain/liver)**	**Urinary copper excretion (*μ*mol/L)**	**CPL (g/L)**	**Copper content in dry liver tissue (*μ*g/g)**	**Liver pathology**	**Leipzig score**	**Treatment; response**
P1	29	Dysarthria, sialorrhea, and paroxysmal dyskinesias in upper and lower extremities	Yes	Dysarthria, Parkinsonian syndrome, sialorrhea	T2 hyperintensities in the dorsal pons, mesencephalon, medial cerebellar peduncles, thalamus and putamen	5.2	0.03	887	Cirrhosis in biopsy	10	Penicillamine; partially stabilized without marked improvement
P2	12	Abnormal biochemical liver tests initially identified during salmonella enteritis infection (ALT 5x ULN, AST 3x ULN)	No	Asthenia, hypermobility, muscle hypotonia, arterial hypertension, subclinical hypothyroidism	Hepatomegaly, advanced diffuse liver parenchyma findings of fibroinflammatory process	2.6	0.08	Liver biopsy not performed	Cirrhosis. Moderate steatohepatitis by ultrasound and MRI	5	Penicillamine; fully stabilized on the treatment
P3	14	Elevated liver tests (ALT 2xULN), accidentally found	No	Normal	Not performed	1.2	0.06	1302	Hepatitis and steatosis in biopsy	6	Penicillamine; fully stabilized on the treatment
P4	5	Elevated liver tests, accidentally found	No	IBS	Not performed	2.0	0.08	731	Steatosis, fibrosis in biopsy	5	Kelatine; marked improvement in Cu excretion

The study was approved (#42/23) by the Institutional Review Board of the First Faculty of Medicine, Charles University, Prague.

Written informed consent was obtained from each patient included in the study. The study protocol conforms to ethical guidelines of the 1975 Declaration of Helsinki.

### 2.1. Sanger Sequencing and MLPA Assay of *ATP7B*

gDNA was extracted from peripheral whole blood samples (WBC) by a standard protocol. Coding (and promoter) regions (P1:c.‐1145 and P3:c.‐273; NM_00053.4) of *ATP7B* (NG_008806.1) were amplified by PCR from gDNA. Sanger sequencing was performed using a 3500xL Genetic Analyzer (Thermo Fisher Scientific).

Large deletions/duplications in *ATP7B* in P1–P3 were excluded using the MLPA assay; SALSA MLPA Probemix P098 WD (MRC Holland, Amsterdam, The Netherlands).

### 2.2. Nasopharyngeal Swab Collection

Sterile nylon swab sticks were used to collect nasopharyngeal swabs. The swab stick was gently inserted through the nostril parallel to the palate until it reached the (naso)pharyngeal fornix. Using a circular motion, the swab stick was rotated several times to maximize cell collection from the nasopharyngeal mucosa (Figure 1a). Immediately after sampling, the swab stick was immersed in the protective agent RLT from the RNeasy Mini Kit (Qiagen, Hilden, Germany) for preservation.

Figure 1Nasopharyngeal swab collection. RNA sequencing data demonstrate abundant expression of *ATP7B* mRNA in nasopharyngeal swabs covering all 21 exons of the longest *ATP7B* transcriptional isoform (NM_000053.4). (a) Schematic of the swab sample collection with a nylon stick from (naso)pharyngeal fornix. (b) The box plot shows the highest *ATP7B* gene expression in nasopharyngeal swab samples, compared to liver, fibroblasts, and WBCs. The abundance is expressed as TPM (transcripts per million). TPM values were calculated based on RNA‐seq data. The dots represent individual samples (*n* = 10) for each tissue. The dashed line represents the threshold TPM = 1, indicating feasibility of PCR analysis. (c) Representation of *ATP7B* isoforms in the nasopharyngeal swab and liver. Comparable profiles of the isoforms in the tissues demonstrate that the nasopharyngeal swab is a suitable material for *ATP7B* mRNA analyses. The main isoform (NM_000053.4; ENST00000242839.10) is the longest and predominant in both tissues. The gray color represents isoforms detected in liver, the blue color represents isoform detected in nasopharyngeal swab samples, and the gray boxes with blue border represent isoforms detected in both tissues. Transcript containing the alternative Exon 1b was detected exclusively in nasopharyngeal swabs. The blue asterisks denote the nasopharyngeal swab transcripts with Exon 1b occurrence 1:1 to the standard exon 1. ENST00000400366.6 was detected exclusively in the liver.

**Figure:**
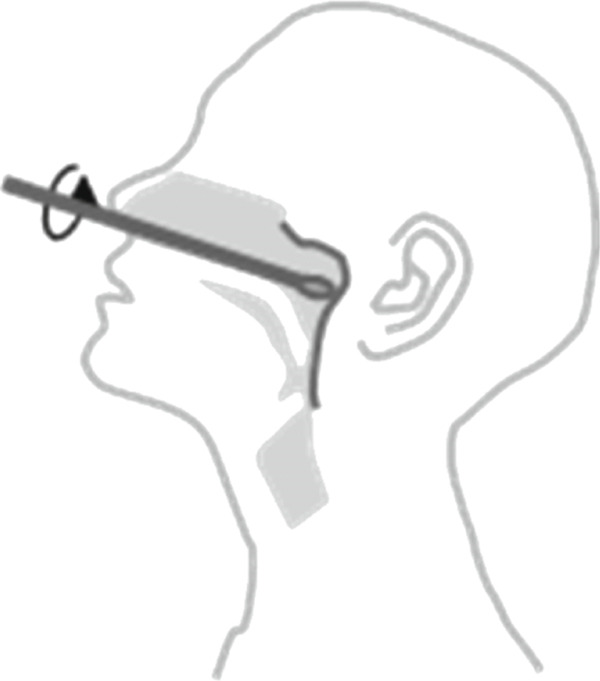
(a)

**Figure:**
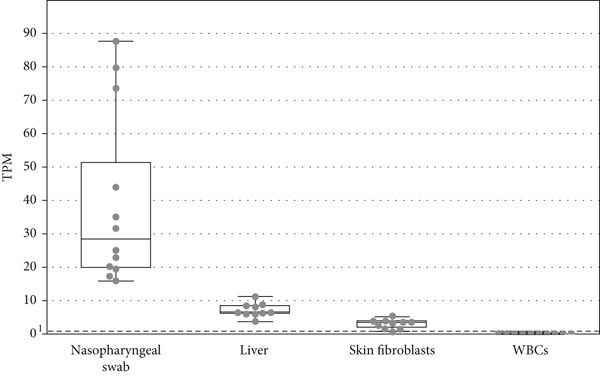
(b)

**Figure:**
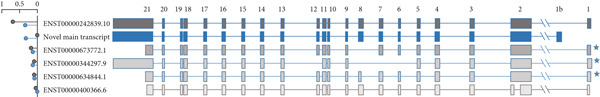
(c)

### 2.3. RNA Sequencing in Control Nasopharyngeal Swabs and Other Somatic Tissues

Total RNA was isolated from nasopharyngeal swabs, liver, fibroblasts and WBCs of control individuals (*n* = 10) using RNeasy Mini Kit. Then, 1 *μ*g of total RNA was further used to prepare stranded rRNA depleted RNA‐seq libraries by KAPA RNA HyperPrep Kit with RiboErase for Illumina platforms (F.Hoffmann‐La Roche, Basel, Switzerland). Paired‐end 2x100 bp reads were generated using NovaSeq6000 (Illumina, San Diego, CA, United States) at the National Center for Medical Genomics in Prague according to the manufacturer′s protocol. The resulting FASTQ files were subjected to quality control, trimmed using Atropos (1.128) [27], and aligned to the human genome reference sequence hg19 using STAR (2.7.8a) with default parameters. Duplicate removal was performed using Picard Tools (2.20.81.129).

Estimation and representation of the transcript isoforms and their exon composition was calculated based on the count of splice junctions. The data were visualized in Integrative Genomics Viewer (2.3; IGV, [28]) as a Sashimi plot, and the number of reads spanning specific splice junctions was put into ratios.

### 2.4. Long‐Range PCR (LR‐PCR) *ATP7B* cDNA Amplicon (*ATP7B_E3-E21*) From Nasopharyngeal Swabs

cDNA was reverse transcribed from nasopharyngeal swab‐isolated total RNA using oligo(dT) and SuperScript IV Reverse Transcriptase (Thermo Fisher Scientific). *ATP7B* cDNA was PCR amplified using primers 5 ^′^‐GTGCTGGGAATTCCATGGTG‐3 ^′^ and 5 ^′^‐TTGTGGTGAGTGGAGGCAAG‐3 ^′^ and a Phusion HotStart Flex DNA Polymerase (New England Biolabs).

A PCR protocol was used: Initial denaturation at 98°C for 30 s was followed by 30 cycles of 98°C for 15 s, 68°C for 20 s, extension at 72°C for 2 min (+ 2 s/cycle) and final extension at 72°C for 5 min, generating 3130‐bp PCR product.

### 2.5. LR‐PCR *ATP7B_I4-I9* Amplicon From gDNA

Genomic region spanning *ATP7B* introns 4 to 9 (*ATP7B_I4_I9*) was PCR amplified from gDNA in P1–P3 using the following primers: the forward with T7 overhang 5 ^′^‐AATACGACTCACTATAGGGCTTTCACAGGCTTTCCT‐3 ^′^ and the reverse with RP overhang 5 ^′^‐CAGGAAACAGCTATGACGATGCAGCTCACACAGATTGA‐3 ^′^.

Two‐step PCR protocol with TaKaRa LA Taq DNA Polymerase with 10x LA PCR Buffer II (TaKaRa, Mountain View, CA) was used: initial denaturation at 94°C for 1 min was followed by 30 cycles of 98°C for 10 s, 57°C for 7 min elongation and final elongation was at 72°C for 10 min generating 7 634 bp PCR product.

### 2.6. Long‐Read Sequencing of *ATP7B_E3-E21* cDNA Amplicons

*ATP7B_E3-E21* amplicons were purified using magnetic beads SPRI (Beckman Coulter, Inc., Brea, CA, United States) according to the manufacturer′s protocol. Beads‐purified PCR products were quantified using Qubit 2.0 Fluorometric Quantitation (Beckman Coulter, Inc.) on Qubit™ 1x dsDNA HS Assay Kit (Thermo Fisher Scientific).

Samples were sequenced using the MinION Oxford NANOPORE platform (Oxford Science Park, United Kingdom) according to the manufacturer′s protocol and Ligation sequencing amplicons—Native Barcoding Kit 24 V14 and Flongle Flow Cell (R10.4.1).

FASTQ files were aligned to the human genome reference sequence (hg19) using minimap2 (v. 2.24) in splice mode and subsequently converted to *.bam* format and sorted using Samtools (v1.15.1). Reads were visualized in IGV.

### 2.7. Long‐Read Sequencing of *ATP7B_I4-I9* gDNA Amplicons

LR‐PCR *ATP7B_I4-I9* amplicons were purified using the protocol for *ATP7B_E3-E21* amplicons. Samples were sequenced on Pacific Biosciences (Menlo Park, CA, United States) Sequel I system according to the manufacturer′s protocol using SMRTbell Express Template Kit 2.0 and Sequel Sequencing Kit 3.0.

To obtain highly accurate reads, circular consensus sequence (CCS) analysis was performed using SMRT Link (v6.0). CCS reads were aligned to hg19 using minimap2 (v2.24), converted to *.bam* format, and sorted using Samtools (v1.15.1). Reads were visualized in IGV.

### 2.8. *In Silico* Prediction of the Splicing Abnormalities and Putative Effects on ATP7B Protein

Splicing effects of identified synonymous variants NM_000053.4(*ATP7B*): c.1488C>T (p.(Gly496=)), c.2241C>T (p.(Ile747=)), c.2292C>T (p.(Phe764=)) [22], and one nonsense variant c.2336G>A (p.(Trp779Ter)) on *ATP7B* mRNA and alterations of exon splicing regulatory elements, including exon splicing enhancers (ESEs) and exon splicing silencers (ESS), were analyzed using the Human Splicing Finder Pro Version: 4.3.3 (HSF Pro; Mutations Analysis Version 2.05) [29].

Homology model of ATP7B protein (NP_000044) was built using program Modeller [30] and Protein Data Bank [31] entry 7SI3 as a template.

### 2.9. Isoform and Allelic Expression Ratios of *ATP7B* Based on Analyses of *ATP7B_E3-E21* Amplicons

Individual *.sam* (Sequence Alignment/Map) *ATP7B*_E3_E21 files of the patients and controls were processed to assign sequencing reads into groups based on their features (e.g., group “full E3E21” contained all 3–21 *ATP7B* exons). The number of reads in individual groups was calculated to determine *ATP7B* transcript isoform ratios. Only continuous reads of *ATP7B_E3-E21* amplicons encompassing Exons 3 and 21 (binding sites of amplification primers) were evaluated. In patient *.sam* files, we simultaneously tracked the allele‐specific variant to determine the phasing and to calculate the allelic expression ratios. Allele‐specific variants were at positions chr13:52511772 (c.3741_3742dup) for P1, chr13:52518281 (c.3207C>A) for P2 and P4, and chr13:52523808 (rs732774) for P3. The resulting ratios were manually inspected in IGV.

## 3. Results

### 3.1. Initial Incomplete Genetic Diagnosis in WD Patients P1–P4

Routine Sanger sequencing revealed one previously reported pathogenic variant (further designated as Allele 1) and a second synonymous variant (further designated as Allele 2) in *ATP7B* in each of the four patients (Table 2). Allele 2 variants were annotated in the ClinVar database either as of “conflicting pathogenicity” (c.2292C>T, p.(Phe764=)) in P1 and P2 or as “likely benign” (c.2241C>T, p.(Ile747=)) in P3. Population frequencies of both variants were very low (7.12e‐6 for p.(Phe764=) or zero for p.(Ile747=)) in the gnomAD database; the variant c.1488C>T (p.(Gly496=)) in P4 was unique, and we uploaded the information into the ClinVar database (ClinVar ID 3366313). The phase of the identified *ATP7B* variants was unknown in P1. In P2, P3, and P4, the *trans* phase of the variants was established by parental transmission.

**Table 2:** Genetic findings in the *ATP7B* gene. List of *ATP7B* variants identified in WD patients P1–P4 initially diagnosed with one heterozygous pathogenic *ATP7B* variant (Allele 1 (A1)). Synonymous Allele 2 (Allele 2 (A2)) variants are highlighted in bold. The initial aggregate germline classification for identified variants in the ClinVar database is listed.

**Patient ID**	**Allele**	***ATP7B* genetic lesions** **Reference NM_000053.4**	**Exon location**	**gnomAD frequency/ClinVar ID/classification**
P1	A1	c.3741_3742dup (p.Lys1248fs)	18	0.000007118/1456045/pathogenic
	**A2**	**c.2292C>T (p.Phe764=)**	8	0.000007118/157937/conflicting classifications of pathogenicity
P2	A1	c.3207C>A (p.His1069Gln)	14	0.001019/3848/Pathogenic
	**A2**	**c.2292C>T (p.Phe764=)**	8	0.000007118/157937/conflicting classifications of pathogenicity
P3	A1	c.2336G>A (p.Trp779Ter)	8	0.00003917/156284/pathogenic
	**A2**	**c.2241C>T (p.Ile747=)**	8	0/1133934/Likely benign
P4	A1	c.3207C>A (p.His1069Gln)	14	0.001019/3848/Pathogenic
	**A2**	**c.1488C>T (p.Gly496=)**	3	0/None/None

*In silico* prediction analyses using the HSF software indicated that the synonymous variants c.1488C>T (p.(Gly496=)), c.2292C>T (p.(Phe764=)), and c.2241C>T (p.(Ile747=)) may affect ESE and ESS motifs and could lead to altered splicing of *ATP7B* mRNA. In contrast, the c.2336G>A (p.(Trp779Ter)) variant did not affect exonic splicing ratio (ESR) profiles (Figure 2a).

Figure 2Visualization of *in silico* HSF predictions for studied *ATP7B* variants. *ATP7B_E3-E21* amplicon sequencing data in control tissues compared to WD patients show deleteriously increased Exon 8 skipping due to variants identified in Patients 1–3. (a) ESR (ESE/ESS ratio) profiles generated by the HSF predictor compare the distribution of splicing enhancers and silencers. Analyzed *ATP7B* variants are in red in the sequences above the ESR profiles. ESR profiles of sequences containing the analyzed variants are in red, while profiles for the reference sequence are in green. The decreases in the score suggest aberrant splicing caused by the three synonymous variants (red lines). Prediction for the p.(Trp779Ter) variant did not indicate any changes in the ESR profile (only the green line is present). (b) The schematic represents the most abundant *ATP7B* isoforms. Red boxes highlight the skipping events in the particular transcripts. Amplicon sequencing data in controls (top right) show a similar occurrence of *ATP7B* isoforms in nasopharyngeal swabs (*n* = 20) and liver tissues (*n* = 7). Solid color‐filled bars represent amounts of complete E3E21 isoform encoding full functional protein (applies for synonymous variant), while white filled bars highlight groups of transcripts with naturally occurring exon skipping events (d8, d8_144b, and d6d7d8). (c) WD patients P1, P2, and P3 produce predominantly mutant (p.His1069Gln) or *ATP7B* transcripts with skipped exons (d8 and d6d7d8). The transcripts with skipped exons (d8, d8_144b, and d6d7d8) originate mainly from Allele 2 bearing the synonymous (P1 and P2) or the nonsense (P3) variants.

**Figure:**
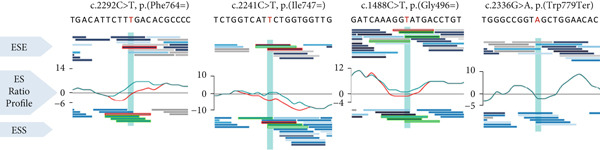
(a)

**Figure:**
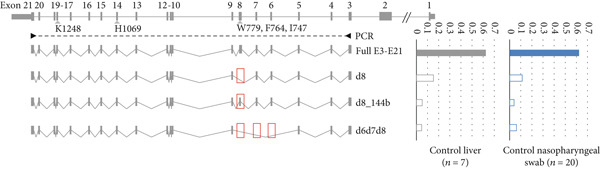
(b)

**Figure:**
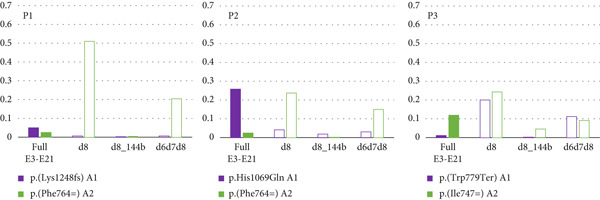
(c)

### 3.2. *ATP7B* mRNA Is Abundantly Expressed in Nasopharyngeal Swabs

We performed RNA sequencing in control (*n* = 10) nasopharyngeal swabs, liver, fibroblasts, and WBCs and calculated the TPM (transcripts per million) values. The highest abundance of *ATP7B* mRNA was detected in nasopharyngeal swabs (TPM = 39.5), lower in liver (TPM = 7.4), skin fibroblasts (TPM = 3.1), and negligible in WBCs (TPM = 0.3) (Figure 1b).

*ATP7B* mRNA profile in nasopharyngeal swabs was comparable to the profile identified in the liver. Both were mainly composed of transcripts containing all 21 exons corresponding to the major reported isoform (NM_000053.4, ENST00000242839.10; Figure 1c). We identified novel alternative Exon 1b, located on chr13: 52,569,450–52,569,854 (hg19), with putative start (ATG) codon (chr13: 52,569,464), exclusively in nasopharyngeal swab samples. The abundance of the alternative Exon 1b is similar to the conventional Exon 1 in a ratio ~1:1. The main isoform ENST00000242839.10 in liver and in nasopharyngeal swabs (starting by Exon 1 or Exon 1b) represents about 70% of all *ATP7B* transcripts. Isoforms ENST00000673772.1 and ENST00000344297.9 missing Exon 8 and Exons 6, 7, 8, and 12 (Figure 1c) comprise about 5%–15% of all transcripts. Isoform ENST00000400366.6, which lacks part of Exon 2, was detected only in liver in ~1% of all transcripts.

### 3.3. Long‐Read Sequencing of *ATP7B* cDNA From Nasopharyngeal Swabs Established Diagnosis in Four Genetically Unresolved WD Patients

We PCR amplified a region spanning from Exon 3 to 21 (*ATP7B_E3-E21* amplicon) from liver (*n* = 7) and nasopharyngeal swab (*n* = 20) samples of control individuals and from nasopharyngeal swabs of patients P1–P4. The amplicons were sequenced using the Oxford Nanopore sequencing platform.

In controls, 62% of all transcript molecules from nasopharyngeal swab and liver corresponded to the major full *ATP7B* transcript (ENST00000242839.10) comprising Exons 3–21. Then, 11% (nasopharyngeal swab) and 15% (liver) corresponded to the second most abundant *ATP7B* transcript lacking Exon 8 (d8, ENST00000673772.1). Then, 4% (nasopharyngeal swab) and 5% (liver) were transcripts lacking only 144 bases from the 5^′^ region of Exon 8 (d8_144b, ENST00000634844.1). The fourth most frequent group of *ATP7B* transcripts was a group with Exon 6‐7‐8 skipping (d6d7d8) making up to 6% (nasopharyngeal swab) and 5% (liver) (Figure 2b). Other less frequent groups (< 2%) of transcripts were d6d7, d12, d8d12, and d5d6d7d8.

In Patient 1 (Figure 2c), only a small fraction of transcripts (7%) originated from Allele 1 (p.(Lys1248fs)). Among transcripts from Allele 2 (p.(Phe764=)), transcripts with Exon 8 skipping predominated (51% d8 and 21% d6d7d8). Overall in Patient 1, only 3% of the transcripts were the full E3E21 transcript putatively encoding functional ATP7B protein.

Patient P2 (Figure 2c) generated 31% of full E3E21 *ATP7B* transcripts from Allele 1 (p.His1069Gln). d8, d8_144b, and d6d7d8 transcripts from this Allele 1 were detected at the same rate as in controls. From Allele 2 (p.(Phe764=)), a substantially larger fraction of d8 and d6d7d8 (24% and 15%) transcripts were identified. In Patient 2 (similar to Patient 1), only 2% of the *ATP7B* transcripts were full E3E21.

Patient P3 (Figure 2c) Allele 1 (p.(Trp779Ter)) produced mainly d8 and d6d7d8 isoforms (20% and 11%). Allele 2 (p.(Ile747=)) produced similar fractions of d8 and d67d8 transcripts (24% and 9%). Unlike d6d7d8 transcripts, the percentage of d8_144b transcripts from Allele 2 was slightly higher than from Allele 1. Overall, the full E3E21 isoforms putatively encoding functional protein constituted only 13% (A1: 1% and A2: 12%) of all *ATP7B* transcripts.

Patient P4 generated 45% of *ATP7B* transcript molecules from Allele 1 (p.His1069Gln). The remaining 55% of the transcript molecules originated from Allele 2 (p.(Gly496=)). Importantly, all of the Allele 2 transcript molecules carried a unique partial Exon 3 deletion (del3_57b, chr13:52,544,628‐52,544,684; NM_000053.4:c. 52544628_5,5446840del, hg19).

Other rare variants potentially contributing to exon 8 skipping were excluded in P1, P2, and P3 by long‐read sequencing of the genomic region spanning introns 4 to 9 (*ATP7B_I4-I9*) of the *ATP7B* gene on gDNA (Table 3).

**Table 3:** List of SNPs identified by LRAS of the *ATP7B_I4-I9* region on gDNA in patients P1–P3. Genotyping of the *ATP7B* region spanning Introns 4–9 excluded any rare variants that additionally contributed to Exon 8 skipping (del8).

**Genomic coordinate**	**Reference allele**	**Variant allele**	**Zygosity**	**Location**	**gnomAD allele frequency**
13:52531960	C	T	Heterozygous	Intron 8	*ƒ* = 0.564
13:52532039	T	C	Heterozygous	Intron 8	*ƒ* = 0.376
13:52536266	T	C	Heterozygous	Intron 5	*ƒ* = 0.540
13:52538664	C	G	Homozygous	Intron 5	*ƒ* = 0.939

Long‐read sequencing revealed the *trans* phase of Allele 1 and 2 variants and compound heterozygosity in all four WD patients.

## 4. Discussion

The key novel finding presented in our study is the identification of an abundant expression of *ATP7B* in the material collected by nasopharyngeal swabs. As a practical diagnostic application, we demonstrate that long‐read sequencing analyses performed in these samples allow effective *in vivo* testing/validation of the effects of variants (synonymous included) on *ATP7B* mRNA synthesis and processing in suspected WD patients.

Recently, a new category of genetic variants designated as “unsense” has been proposed [32, 33] to emphasize changes targeting splicing regulation, gene expression, or leading to impaired mRNA splicing due to exonic substitution(s). Such synonymous variants potentially leading to exon skipping have also been recently reported in the *ATP7B* gene in (suspect) WD patients [22–26]. Studies validating pathogenic impacts on mRNA processing in WD patients have been, however, so far limited by (i) low expression of *ATP7B* in WBCs and the need for invasive liver biopsy(ies) or (ii) use of elaborate *in vitro* minigene splicing assays. Identification of a surrogate anatomic sampling site allowing testing of *ATP7B* mRNA is, therefore, highly desirable.

Our data show intriguingly higher expression of *ATP7B* in nasopharyngeal swabs compared to liver (Figure 1b). The overall content and relative abundance of individual *ATP7B* transcripts are comparable between liver and nasopharyngeal swab samples (Figure 2b). The only difference we observed between the two tissues is the novel alternative Exon 1b in a ratio of 1:1 to the conventional Exon 1 that we detected exclusively in the nasopharyngeal swab. At this point, we can only speculate about the implications of these findings for nasopharyngeal mucosal function under normal and pathological conditions. It has been demonstrated [34, 35] that transcription profiles in various endodermal cell types may overlap. High *ATP7B* expression in nasopharyngeal mucosa and cells such as hepatocytes could thus reflect their shared developmental origin despite organ‐specific differences.

As a practical diagnostic application of our observations, we developed an effective protocol for long‐read sequencing of *ATP7B* cDNA amplicons from the nasopharyngeal swab samples. By documenting splicing impacts of synonymous *ATP7B* variants, our novel technique established molecular WD diagnosis in four patients with previous inconclusive results of *ATP7B* standard genetic testing (Tables 1 and 2).

Total fractions of Allele 2 (p.(Phe764=)) major isoform transcripts containing Exon 8 and putatively translating to a functional ATP7B protein were limited to 3% and 2% in Patients 1 and 2, respectively. The majority of Allele 2 transcripts lacked the entire Exon 8 (d8). The rest of the *ATP7B* transcripts in these two patients originated from Allele 1 (p.(Lys1248fs) or p.His1069Gln). The relative paucity of Allele 1 transcripts carrying the p.(Lys1248fs) in Patient 1 suggested nonsense‐mediated mRNA decay resulting from this variant (Figure 2c).

c.2336G> A variant putatively results in a STOP codon (p.(Trp779Ter)). Nonsense mediated mRNA decay and/or protein truncation resulting from this variant has been suggested by a number of authors [36–38]. Waldenström et al. [39], however, hypothesized about the contribution of alternative splicing [40]. We show that the majority of Allele 1 (p.(Trp779Ter)) transcripts in Patient 3 lacked Exon 8 (d8). However, the HSF software did not predict any changes in the ESR profile (Figure 2a). The amounts of transcripts carrying the STOP codon at position 779 were negligible. Similar to Patients 1 and 2, Allele 2 (p.(Ile747=)) major isoform transcripts (full E3E21) constituted only a minor fraction (12%) of all *ATP7B* transcripts in Patient 3 (Figure 2c).

On the protein level we hypothesize that the in‐frame Exon 8 skipping (d8), identified in patients P1, P2, and P3 due to p.(Phe764=), p.(Ile747=), and p.(Trp779Ter), leads to the synthesis of an unstable ATP7B protein lacking transmembrane domains essential for the transport function and proper protein folding (Figure 3).

Figure 3
*ATP7B* Exon 8 skipping is predicted to cause the absence of protein domains essential for the transmembrane transport function and proper protein folding of the ATP7B protein. (a) Upper schematic highlights the individual exons and positions of the detected variants in the *ATP7B* mRNA. The range of the LR‐PCR amplified sequence (Exons 3–21) is depicted below the schematic. Black rectangle corresponds to Exon 8 deletion (d8). Black‐gray inner part of the rectangle corresponds to the d8_144b region. Lower schematic outlines the domain structure of ATP7B (MBD1‐6 ‐ metal binding domains 1‐6; MA, MB and M1–6—membrane domains). Color coding corresponds to ATP7B protein domain structure in (b) and Bitter et al. [41]. Exon 8 skipping (highlighted as a black rectangle) resulting from synonymous variants p.(Phe764=), p.(Ile747=), and a nonsense variant p.(Trp779Ter) leads to an absence of 78 amino acid residues of transmembrane domains M1, M2, and partly MB. Exon 3 skipping (del3_57b) resulting from the synonymous variant p.(Gly496=) in P4 is expected to result in an absence of 19 amino acid residues (in bright green) of ATP7B MBD5. Black dashed line limits the extent of the ATP7B protein model in (b). (b) ATP7B protein model was built based on the cryoelectron microscopy structure of the *Xenopus tropicalis* ATP7B [41]. Positions of the variants are highlighted. Regions of the protein putatively affected by del3_57b and del8 are marked in bright green and black, respectively.

**Figure:**
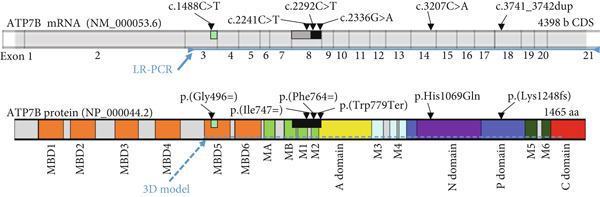
(a)

**Figure:**
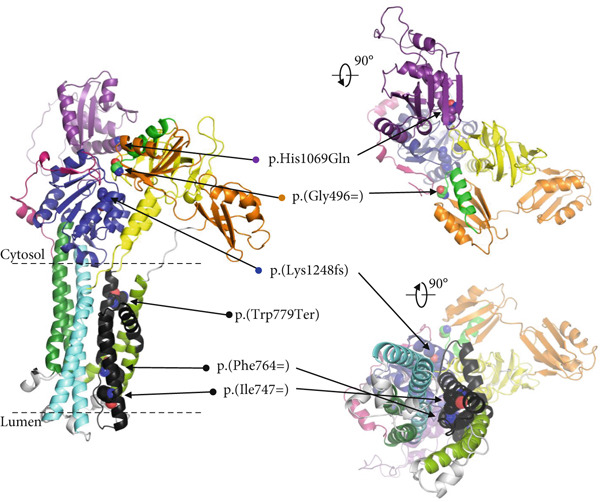
(b)

As demonstrated in control samples of tested tissues (Figure 2b), the region of *ATP7B* Exons 5–9 is rich in exon skipping events (detected in up to 25% of transcripts). Importantly, the synonymous variants detected in Patients 1 and 2 and the nonsense variant detected in Patient 3 impact the milieu of *ATP7B* transcripts, resulting in substantially increased fractions of molecules with skipped exons.

In P4, the allelic expression ratio was approximately 1:1. Allele 1 transcripts carried the pathogenic variant p.His1069Gln, whereas the majority of Allele 2 (p.(Gly496=)) transcripts were missing part of Exon 3 (del3_57b; Figure 2c). This partial in‐frame skipping of Exon 3, due to the unique synonymous variant p.(Gly496=), is predicted to encode ATP7B protein lacking part of the metal‐binding domain 5 (Figure 3).

Altogether, the long‐read sequencing protocol documented only a minimal (up to 13%) fraction of *ATP7B* transcripts translating to a functional protein in nasopharyngeal swabs of all four WD patients. We optimized the method for the *ATP7B_E3-E21* amplicon because of the distribution of the variants in our patients with a focus on Exon 8. Nevertheless, the range of the sequence for long‐read analyses can be individually modified to study the effects of variants outside the *ATP7B_E3-E21* region.

We show that our approach allows (i) identification and quantification of *ATP7B* mRNA isoform diversity and (ii) easy assessment of the phase (*cis* vs. *trans*) of the variants. The latter is particularly helpful when parents are not available for genetic testing. By documenting deleterious effects of p.(Phe764=), p.(Ile747=), and p.(Gly496=), we show that mRNA analyses in nasopharyngeal swabs allow to characterize additional *ATP7B* variants of unknown significance, including rare synonymous variants identified by standard genetic testing. Moreover, long‐read sequencing of the *ATP7B* cDNA amplicons can reveal, either directly or indirectly, the presence of noncoding variants impacting expression, processing, or mRNA stability, as well as structural genetic lesions including larger genomic deletions, duplications, and rearrangements.

Highly effective WD treatments with copper chelators or zinc salts are available to pre‐symptomatic patients with established biallelic *ATP7B* lesions [9]. Strategies of *ATP7B* variant pathogenicity validation such as our protocol, therefore, not only complement other diagnostic methodologies like quantification of ATP7B protein in dried blood spots [42], facilitate genetic counselling in the affected families, but also help to identify individuals potentially benefiting from early therapy initiation.

Moreover, *ATP7B* variants have been reported by association studies as potential contributors to the pathogenesis of conditions such as Alzheimer disease [43, 44] or the efficiency of cascades of chemotherapy response/resistance [45]. Effective assessment of the impacts of *ATP7B* variants may thus have positive sociomedical impacts beyond the community of WD patients and medical specialists. As a last potential practical implication, nasopharyngeal swab material may offer gene transcripts relevant for molecular diagnostics of other monogenic conditions that are, similar to WD, limited by organ‐specific expression patterns.

## 5. Conclusion

Our results demonstrate that synonymous variants, usually excluded from the interpretation of sequencing data, should be perceived with caution and considered as potential critical contributors to WD. Long‐read sequencing of the *ATP7B* mRNA amplicons generated from easily obtainable nasopharyngeal swabs may help to identify splicing impacts and allow phasing of the variants. We are readily available to collaboratively provide the outlined long‐read sequencing analyses in nasopharyngeal swabs of suspect WD patients and share the control RNA sequencing data upon reasonable request.

**Abbreviations**
*ATP7B_E3-E21*
: long‐range PCR *ATP7B* cDNA amplicon encompassing Exons 3–21
*ATP7B_I4-I9*
: long‐range PCR *ATP7B* gDNA amplicon encompassing Introns 4–9
LRAS: long‐read amplicon sequencing
LR‐PCR: long‐range PCR
WBCs: white blood cells
WD: Wilson disease

## Ethics Statement

The study was approved (#42/23) by the Institutional Review Board of the First Faculty of Medicine of Charles University and General University Hospital in Prague, Prague. Written informed consent was obtained from each patient included in the study, and the study protocol conforms to the ethical guidelines of the 1975 Declaration of Helsinki.

## Conflicts of Interest

The authors declare no conflicts of interest.

## Author Contributions

L.S.M., A.V., F.M., L.N., V.S., S.K., J.S. and I.J. were involved in the writing of the manuscript and revised the manuscript. L.S.M., S.K., J.S. and I.J. edited the final version of the initially submitted and revised manuscript. I.J. submitted the manuscript. L.S.M., A.V., F.M., D.Z., I.B., P.D., D.H., and I.J. performed the laboratory experiments and interpreted the data. F.M. and V.S. performed bioinformatic studies. J.M., J.K., J.Š., S.M., M.G., P.S., R.B., and P.D. provided the clinical evaluation of the patients. R.B. and P.D. coordinated clinical data interpretation. I.J. and L.S.M. coordinated the study. L.S.M., A.V., and F.M. contributed equally to this work.

## Funding

This work was supported by the Ministry of Health of the Czech Republic in cooperation with the Czech Health Research Council under projects No. NU23‐07‐00281, NW24‐04‐00067, and RVO‐VFN 64165; National Center for Medical Genomics, LM2023067; National Institute for Neurological Research—Funded by the European Union—Next Generation EU (Programme EXCELES, ID Project No. LX22NPO5107); Next Generation EU through the Recovery and Resilience Plan for Slovakia, 09I03‐03‐V03‐00007; MULTIOMICS_CZ (Programme Johannes Amos Comenius, Ministry of Education, Youth and Sports of the Czech Republic, ID Project CZ.02.01.01/00/23_020/0008540) funded by the European Union–Next Generation EU; and Univerzita Karlova v Praze, 10.13039/100007397, UNCE‐24/MED/022.

## Data Availability

The data that support the findings of this study are available on request from the corresponding author. The data are not publicly available due to privacy or ethical restrictions.
